# Message of Sympathy from the I. H. A. to the Venerable Dr. Wells

**Published:** 1890-08

**Authors:** 


### MESSAGE OF SYMPATHY FROM THE I. H. A. TO THE VENERABLE DR. WELLS.

The following is the text of the message of sympathy which the International Hahnemannian Association, at its annual meeting, directed to be telegraphed to Dr. P. P. Wells, who by reason of serious illness was unable to attend the meeting:

Watch Hill, R. I., June 24th, 1890.

*To P. P. Wells, M. D.*, 158 *Clinton Street, Brooklyn, N. Y.*

Dear and Honored Colleague:—The International Hahnemannian Association hears with profound sorrow of your illness and regrets the loss of your counsel. It desires to convey its deepest sympathy, and hope that the grand art of Hahnemann may afford you both relief of suffering and restoration to health.

Jos. A. Biegler, *President.*

*Committee:* William P. Wesselhœft, Clarence W. Butler, Edward Rushmore.

---

A message of similar tenor was telegraphed to Dr. E. A. Ballard, of Chicago, who has been all his life distinguished for his stalwart maintenance of the homœopathic principle and his devotion to the prosperity of the I. H. A.

